# Prognostic factors in ALS: different approaches to the same problem

**DOI:** 10.1055/s-0045-1809407

**Published:** 2025-06-20

**Authors:** Maria Cristina Vázquez, Abayubá Perna, Mariana Legnani, Gustavo Saona

**Affiliations:** 1 Universidad de la República, Hospital de Clínicas, Departamento de Neurología, Montevideo, Uruguay.; 2 Investigator independiente, Montevideo, Uruguay.

**Keywords:** Amyotrophic Lateral Sclerosis, Uruguay, Survival, Bias, Prognosis

## Abstract

**Background:**

The natural history of amyotrophic lateral sclerosis (ALS), the prognoses, and the survival times are fields of considerable interest that are scarcely studied in South American countries.

**Objective:**

To describe the survival of a representative cohort of Uruguayan ALS patients, and to identify covariates associated with survival using different analyses.

**Methods:**

Survival was assessed using the Kaplan-Meier method. Different Cox proportional hazards functions were used to identify independent prognostic predictors since the diagnosis: classic, stratified, and truncated.

**Results:**

We included 166 definite and probable ALS patients. The median follow-up was of 13.6 years. An analysis was performed according to the recruitment groups: prevalent, exhaustive incident, and non-exhaustive incident cases. The median survival since the diagnosis was longer in the prevalent group (33 months) than in the exhaustive incident (22 months) and non-exhaustive incident (14 months) groups. The median survival time of the entire cohort from onset to death was 37 months and 23 months from the diagnosis. Factors related to survival from diagnosis to death were: age at onset, bulbar region onset, clinical form, and progression rate.

**Conclusion:**

The present study described the role of clinical and demographic factors in ALS survival in the Uruguayan population and shed light on differences involving survival models and the temporal bias produced by the lack of precision in determining the onset of the disease.

## INTRODUCTION

Amyotrophic lateral sclerosis (ALS) is a neurodegenerative disorder characterized by upper and lower motor neuron degeneration. It features considerable clinical heterogeneity in its phenotypic presentation, as well as in the survival. 1 2 3 Older age, bulbar onset and rate of symptom progression have been consistently reported as leading to a worse outcome. 4

More recently, models have been developed to provide an individualized prognosis and identified prognostic factors. 5 Relatively few studies 6 7 8 9 have been performed in South American about survival and prognostic factors due to difficulties in epidemiological data retrieval. A population-based study about the incidence and prevalence of ALS in Uruguay has been conducted, 10 but with no analysis of these factors. The presence of censored survival times to the right in the follow-up of ALS patients is well known. However, the consequences of diagnostic delay and inaccurate knowledge of the onset of the disease have not been taken into account so far.

**Figure 1:**
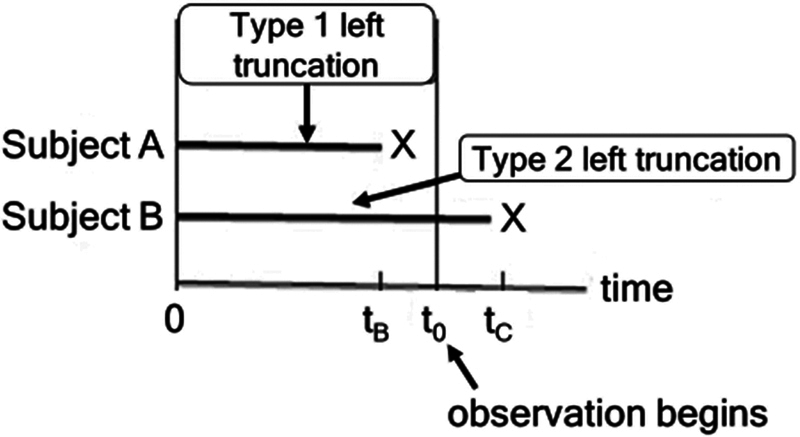
Types of left truncation. Type-I left truncation can affect the representativeness of the sample, while type-II affects the exposure time. 11

These characteristics affect the follow-up time from the left and constitute a non-random selection that produces a truncation bias on the left. The consequence of left truncation is a bias in the inclusion of patients or the observation of a shorter follow-up time. 11 ( Figure 1 )

The aim of the present study is to describe survival of a cohort of ALS patients in Uruguay and to evaluate the prognostic role of selected clinical indicators by exploring statistical analysis strategies.

## METHODS

The current is a single-cohort observational study that included 166 patients with a diagnosis of probable or definite ALS, between January 1, 1996, and December 1, 2006.

From January 1, 2002, until December 31, 2003, a prospective study was conducted considering the entire Uruguayan population and included patients with probable or definite ALS. Multiple sources of recruitment (neurologists, neurophysiologists, hospital registries, the pharmaceutical industry, and death certificates) were used. Some of the cases were prevalent, with a diagnosis of the disease prior to 2002, while others were incident cases diagnosed between 2002 and 2003. In the remaining time, between 2004 and 2006, only patients with ALS referred to the neuromuscular center of our hospital were included, which were all incident cases.

As aforementioned, recruitment began in 2001, including patients diagnosed with probable or definite ALS according to the revised El Escorial criteria. In all cases, the patients were examined and followed up prospectively by neurologists from the ALS research team to confirm the diagnosis. No primary lateral sclerosis or progressive muscular atrophy cases were included in the study. The study was approved by the Ethics Committee at the Teaching Hospital, School of Medicine, Universidad de la República, Uruguay.

The ALS patients were divided into 3 groups: group 1 included patients with diagnosis before 2002 and who were alive on January 1, 2002 (prevalent cases); the patients in group 2 were diagnosed between 2002 and 2003, with the exhaustive uptake mentioned within the entire national population (exhaustive incident cases); and group 3 included patients diagnosed with ALS from January 1, 2004 to December 1, 2006 who were referred for assessment to the group of neuromuscular diseases at our hospital (non-exhaustive incident cases).

Informed consent was obtained from all patients. During December 2016, the dates of death of the patients were updated through telephone calls to their relatives, and death certificates were reviewed.

The prognostic variables studied were: age, sex, region of onset (bulbar or spinal), predominantly upper motoneuron (UMN) or lower motoneuron (LMN) signs, time of diagnostic delay (time between onset of symptoms and diagnosis of disease), score on the Amyotrophic Lateral Sclerosis Functional Rating Scale (ALSFRS), riluzole treatment, and disease progression.

The rate of progression was calculated as the ratio of (40 - ALSFRS at the time of first examination)/(time period from the symptomatic onset at the time of first examination in months). Survival analysis was performed using the Kaplan–Meier method, the log-rank test, and the Cox model.

Different survival multivariate models were applied:

- Model I, or classic analysis: follow-up was considered as starting on the date of diagnosis; patients are at risk of dying from the diagnosis.
- Model II, or stratified analysis: follow-up was considered as starting on the date of diagnosis, but the model was stratified by diagnostic delay (time between onset of symptoms and diagnosis, categorized in quartiles).
- Model III, or analysis considering truncation and risk of death from the onset of symptoms: follow-up was considered as starting since the onset of symptoms, and the model also considers the diagnosis date to enroll patients in the study.
- Model IV, or analysis considering truncation and risk of death since birth: follow-up was considered as starting since birth, and the model also considers the date of diagnosis to enroll patients in the study.

Model II does not directly consider the diagnostic delay, but it differentiates the presence of patients with a longer evolution time of the disease.

Models III and IV consider the truncated nature of ALS follow-up, because the time before diagnosis is generally underestimated and not incorporated into the analysis. 11 We have decided to compare analysis strategies with risk origin at the onset of symptoms or at birth, but with study entry on the date of the diagnosis.

The variables included in the Cox model were chosen according to statistical and clinical criteria. The median follow up period was calculated through the reverse Kaplan-Meier method. 12 A significance level of 5% was adopted for the statistical tests. Calculations were performed using the Stata 16.1 software (StataCorp LLC).

## RESULTS

Our cohort included 166 patients, 38% of them women. The mean age at onset was of 58.7 ± 12.4 years. The site of spinal onset was identified in 67% of the sample. Predominant LMN involvement was observed in 67.5% of the patients. There were 6 cases of familial ALS (3.6%). The median diagnostic delay in months was of 10.13 and the median overall follow-up was of 13.6 years.

**Figure 2:**
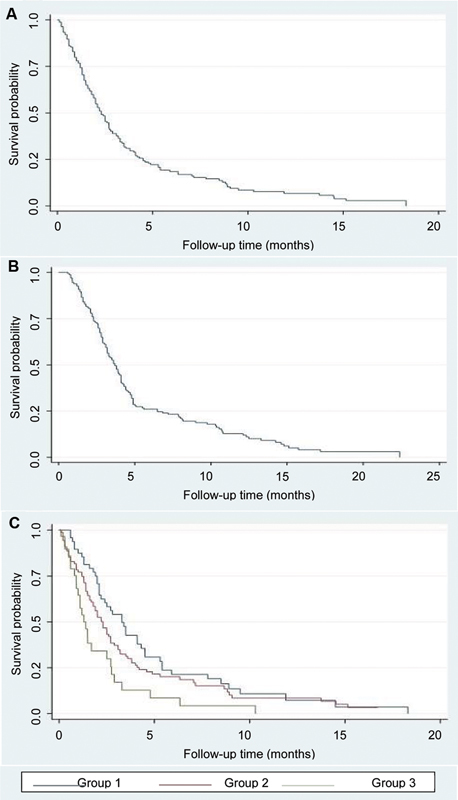
( **A** ) Overall Kaplan-Meier survival estimates (survival since diagnosis); ( **B** ) overall Kaplan-Meier survival estimates (survival since symptom onset); ( **C** ) Kaplan-Meier survival estimates (since diagnosis) according to the recruitment group.

As for the survival analysis, Figure 2A shows the overall survival of the sample since diagnosis, which was of 23 months; and Figure 2B shows the overall survival since symptomatic onset, which was of 37 months. When calculating survival medians by subgroups within the cohort ( Figure 2C ), group 1 (prevalent cases) presented a survival of 33 months, while group 2 (exhaustive incident cases), of 22 months, and group 3 (non-exhaustive incident cases), of 14 months. The difference was statistically significant ( *p* = 0.007) regarding the calculation since the date of the diagnosis.

No statistical differences were found for survival when comparing the men and women in the cohort using the bivariate analysis of survival and considering the time since diagnosis ( *p* = 0.76).When analyzing survival according to age groups, subjects younger than 45 years presented a statistically significant higher survival ( *p* = 0.033). The median survival was of 41 months for patients younger than 45 years, of 22 months for those aged between 45 and 64 years, and of 18 months for the group aged 65 years or older.

According to the site of onset, patients with spinal forms presented a longer survival compared to those with bulbar forms, which was statistically significant (24 versus 21 months respectively; *p* = 0.043). Cases of predominant UMN involvement presented a longer median survival compared with patients with predominant LMN features (33 versus 20 months respectively; *p* = 0.035). There was no statistically significant difference in survival between patients treated or not with riluzole. When assessing survival according to diagnostic delay (longer or shorter than 10 months), no statistically significant difference was found between the two groups ( *p* = 0.059).

The median progression rate for the entire cohort was of 0.8. Therefore, when analyzing the survival rate according to the progression rate, an inverse relationship is described: 31 months for those with a lower progression rate and 15 months for those with a higher rate ( *p* = 0.001). Progression rate, riluzole use, and survival were the only variables with statistically significant differences in the analysis of the three subgroups described ( Table 1 ). Table 2 presents the results of the Cox models for the different multivariate analysis strategies.

**Table 1:** Comparison of the clinical and demographic variables of the three groups of patients

Factor	Group 1 (n = 48)	Group 2 (n = 88)	Group 3 (n = 30)	Total (n = 166)	*p* -value
Female sex % (n)	39.6% (19)	34.1% (30)	46.7% (14)	38.0% (63)	NSD ^μ^
Mean age at onset (years)	57.9 ± 13.1	59.3 ± 12.2	58.4 ± 12.3	58.7 ± 12.4	NSD ^χ^
Bulbar onset ^#^ % (n)	25% (12)	33% (29)	46.7% (14)	33.1% (55)	NSD ^μ^
Lower motoneuron ^##^ % (n)	68.9% (31)	67.1% (57)	66.7% (20)	67.5% (108)	NSD ^μ^
Riluzole treatment: % (n)	52.1% (25)	11.4% (10)	10.0% (3)	22.9% (38)	< 0.0001 ^μ^
Mediuan diagnostic delay (months)	12.7	10.13	9.65	10.13	NSD ^€^
Median progression rate	1.1	0.7	1.3	0.8	0.043 ^€^
Median survival since diagnosis (months)	32.9	22	14	23	0.007 ^★^

Abbreviation: NSD, no significant difference.

Notes: ^#^ Bulbar versus spinal onset; ^##^ lower motoneuron versus upper motoneuron presentation; ^χ^ analysis of variance; ^μ^ Chi-squared test; ^€^ Kruskal-Wallis test; ^★^ Kaplan–Meyer method.

**Table 2:** Results of the Cox models for the different analysis strategies

	Model I		Model II		Model III		Model IV	
	Hazard ratio; 95%CI	*p* -value	Hazard ratio; 95%CI	*p* -value	Hazard ratio; 95%CI	*p* -value	Hazard ratio; 95%CI	*p* -value
Female sex	0.74; 0.50–1.10	0.14	0.77; 0.51–1.16	0.20	0.70; 0.47–1.04	0.078	0.83; 0.53–1.29	0.405
Age at onset	1.01; 1.00–1.03	0.08	1.02; 1.00–1.03	0.04	1.01; 0.99–1.03	0.147	1.04; 0.96–1.12	0.344
Bulbar onset	1.46; 0.97–2.20	0.07	1.57; 1.01–2.46	0.04	1.63; 1.07–2.48	0.024	1.26; 0.79–2.02	0.335
Lower motoneuron	1.68; 1.11–2.55	0.01	1.65; 1.06–2.58	0.02	1.56; 1.02–2.38	0.038	1.59; 1.02–2.49	0.041
Riluzole treatment	0.65; 0.39–1.07	0.09	0.50; 0.29–0.85	0.01	0.57; 0.34–0.95	0.031	0.75; 0.43–1.31	0.316
Progression rate	1.25; 1.14–1.37	< 0.01	1.40; 1.23–1.59	< 0.01	1.39; 1.24–1.55	< 0.01	1.22; 1.07–1.39	0.004

## DISCUSSION

The data herein presented are the first ever produced on survival of a representative cohort of Uruguayan ALS patients.

The limitations of the present study include the combination of incident and prevalent cases, as well as the periods of exhaustive and non-exhaustive uptake of patients. These factors could constitute biases.

The main strengths are the number of patients included, taking into account that ALS is a disease with low incidence and Uruguay has a population lower than 3.5 million inhabitants. Most patients were examined and followed up prospectively, and internationally-accepted diagnostic criteria were used, including only patients with defined or probable ALS, with a long follow-up period.

When comparing the three groups of patients, there was no statistically significant difference among them in terms of the following variables: sex, age, site of onset, predominant UMN or LMN features, and diagnostic delay.

The difference in the progression rate of the disease among the groups was also statistically significant. Group 3 presented the highest progression rate, which might be related to the fact that these patients were referred to the neuromuscular center and may present more aggressive forms.

In the case of group 1, there could be a memory bias, in which the symptomatic onset dates were not reliable, and they were run to the right, explaining a higher rate of progression when compared to group 2.

However, when comparing survival medians among the subgroups of patients, the highest survival rate (of 33 months) was presented by the prevalent cases. This could be due to the absence of poor prognostic factors in these patients who were alive at the beginning of the prospective study. The incident cases presented an intermediate survival, similar to that reported for the entire population. Group 3 presented the lowest survival, which could be linked to a more aggressive disease.

The mean age of ALS onset was of 58.7 years, comparable to what is reported in the meta-analysis by Marin et al. 13 In studies conducted in Latin American countries, 9 14 15 16 the mean age at onset was of 47.5 in Mexico, of 53.6 years in Brazil, of 54 in Ecuador, and of 53 years in Cuba, somewhat lower than the mean age in the current study. Consistent with other studies, 17 33% of the patients presented bulbar onset of the disease.

The median diagnostic delay was of 10 months, somewhat shorter than is the ranges reported in the literature, 18 from 8 to 15 months or longer. Several studies 19 have focused on the diagnostic delay in ALS, with little change over the last years, even after the creation of multidisciplinary ALS centers. In the present study, the research team actively sought cases, which may have decreased the diagnosis time.

The median survival was of 37 months since the first symptoms and of 23 months since ALS diagnosis. Some authors compare survival between population studies and in specialized ALS centers: while some 20 do not find statistical differences, others 21 22 report a longer survival in patients cared for in multidisciplinary centers.

A recent study 16 that compares three cohorts of ALS patients in Cuba, Uruguay, and Ireland shows no differences in survival among the populations. Therefore, we can conclude that the general survival values in our population are comparable with those of population-based studies. 23 24 25 In line with other studies, 4 26 a younger age at ALS onset in our patients was significantly associated with better survival. There was no significant difference in the survival between male and female subjects in the present study, which is similar to other reports. 4 Bulbar onset of symptoms and predominant LMN involvement were associated with a shorter survival, which is in agreement with previous population-based studies. 25

Riluzole use did not show statistical difference in the bivariate analysis of survival. Although there are reports 4 27 that the use of riluzole improves mortality by up to 23% and 15% at 6 and 12 months, respectively, other authors 28 have found that riluzole therapy was associated with a survival advantage only in certain stages of the disease.

Borderline significant differences were found in terms of median survival time since diagnosis after stratifying patients according to the median interval between onset and diagnosis. Authors 13 29 have found that a longer delay leads to a better prognosis, especially with diagnostic delays of 12 months or longer. Previous studies, as well as the current one, show that the rate of disease progression appears to be a sensitive clinical prognostic factor in ALS. 30 31

Truncation is a type of incomplete observation described in survival studies, and it is linked to the event that defines the subject's “entry” into the study (onset of the disease). It is completely different from censoring (the other type of incomplete observation in survival studies), in which the event involved is the dependent variable (in this case, death).

We propose that ALS, such as other neurodegenerative diseases, has a particular form of left truncation (type-II truncation), since we do not know the time of the initial event or risk onset. As can be seen in Figure 1 , type-I left truncation can affect the representativeness of the sample, while type-II left truncation affects the exposure time. In the case of a neurodegenerative disease such as ALS, in which the time of onset of the disease is unknown, this type-II truncation affects all cases. 11

Taking into account these previous aspects, different multivariate survival analysis strategies were planned, including two models (III and IV) that consider data truncation. 11 Models I and IV showed similar results, since they coincide in that the predominant LMN features and the rate of progression are the only significant variables. Given that model I does not consider left truncation makes us suspect that model IV does not adequately comply with the correction of temporal information bias. On the other hand, model II, which stratifies diagnostic delay, recognized a greater number of risk predictors, all well known as risk factors. Similarly, model III recognized bulbar onset, the LMN form, riluzole treatment, and progression rate as prognostic factors.

Models II and III seem to better recognize the prognostic factors of mortality in ALS patients, but it is model III that presents a strategy that incorporates the time before the diagnostic date and allows an analysis that considers left truncation.

It is important to highlight that the retrospective collection of data, such as the date of symptom onset and onset site, is a disadvantage of the current study and of many others that analyze the behavior of this disease. Some symptoms may correspond to other medical conditions, due to the coexistence of morbid states, especially in the elderly. The site of the onset of symptoms also has an impact, since those corresponding to the bulbar level are usually noticed before those of spinal origin.

By considering the diagnosis date, one may obtain greater precision regarding different prognostic factors. However, the moment a subject first comes under observation usually does not coincide with the time when the subject becomes at risk of developing the disease of interest. 32

The first ALS symptoms generally appear when a significant part of the motor system has degenerated, and they probably reflect the loss of compensation mechanisms after a presymptomatic period or subtle symptoms that were not recognized or underestimated. 33 Therefore, it is possible that considering the symptomatic onset enables a closer approximation to the initial event with respect to the moment of diagnosis. However, survival analysis studies in ALS generally do not cover the period from the initial event of the disease until the diagnosis.

The multivariate analysis shows that age at onset as a continuous variable loses value as an independent prognostic factor, being only a statistically significant variable when Cox regression is performed stratified by diagnostic delay. Sex continues to be a non-significant variable, as in the bivariate analysis. Regarding the clinical form of presentation, it behaved as an independent prognostic factor in all types of analysis, with shorter survival in cases of predominant LMN involvement. The site of the onset was also a significant variable in terms of survival, especially in the analysis that considers the time since the symptomatic onset. As indicated by other studies, 34 the clinical form is positioned with greater weight in terms of survival with respect to the site of onset, since it is a more reliable factor based on clinical history, neurological examination, and the prospective follow-up of the case.

The use of riluzole appears as a statistically significant prognostic factor in the multivariate analysis, considering the onset and diagnosis dates as well as stratifying by diagnostic delay. The progression rate behaved as an independent prognostic variable in all types of multivariate analysis. Therefore, there are clinical presentations with higher or lower progression beyond the age at onset, clinical features or site of onset of the disease. Hence, the clinical perception is that, beyond the classical prognostic factors, there are others that seem to influence the progression of the disease and that we are not measuring.

We think that bulbar onset and LMN forms are variables associated with a shorter survival, but their impact or the strength of the association between the variables depends on whether the time of symptomatic onset or diagnosis is considered, given that the diagnostic delay in the cases of bulbar onset and LMN forms is significantly shorter.

The same thing occurs with UMN forms of bulbar onset, which usually present with a prolonged symptomatic history prior to the diagnosis of ALS, with the survival analysis being different if the onset of symptoms or the date of diagnosis is considered as the starting point.

We can conclude then that different analysis strategies enable us to observe that, while some prognostic variables, such as age at onset, use of riluzole, and site of onset, presented greater variation; others were systematically retained, as the motoneuronal clinical form and the rate of progression, which showed a great strength of association with survival in ALS.

Considering the difficulty in measuring time intervals in the diagnostic pathway of ALS, prospective studies will ideally be performed in order to more accurately measure the diagnostic delay. However, even in these types of studies the diagnostic delay remains high, being a problem inherent to those neurodegenerative diseases that require rigorous diagnostic criteria and do not have diagnostic biological markers.
